# Perception of HIV/AIDS Education at the Community Level in Jordan

**Published:** 2017-03

**Authors:** Fathieh ABU MOGHLI, Suhair AL HABEESH, Lubna ABU SHIKHA

**Affiliations:** 1. Dept. of Community Health Nursing, Faculty of Nursing, University of Jordan, Amman, Jordan; 2. Dept. of Nursing, Faculty of Nursing, Al-Zaytoonah University of Jordan, Amman, Jordan; 3. Dept. of Maternal and Child Health, Faculty of Nursing, University of Jordan, Amman, Jordan

**Keywords:** HIV/AIDS education, Attitudes, Students, Jordan

## Abstract

**Background:**

The control of spread of HIV takes concerted efforts at both national and international levels. Education is an important component of preventing the spread of HIV. This study aimed to assess the attitudes of parents, teachers and students towards informing children about HIV/AIDS, attitudes concerning ‘proper’ age to learn about HIV/AIDS, possible differences in attitudes relating to gender of child and what they should learn and ideas about the most ‘adequate’ person/institution to be responsible for provision of HIV/AIDS education.

**Methods:**

This study was conducted in Amman, Jordan in April 2015. Descriptive correlational design was used; a sample of school students, university students, school teachers and parents, a stratified random sample was used. Data was collected by using a questionnaire.

**Results:**

All groups asserted the importance of HIV/AIDS education and awareness rising for all. 62.0% of respondents thought that school was the main source of information. About 82% of respondents believed that HIV/AIDS education should be integrated into different disciplines of school curricula, 84% of respondents believed that HIV/AIDS education should be part of university curricula. Nobody believed that HIV/AIDS education should be restricted to boys only.

**Conclusion:**

As HIV/AIDS is a scary matter to all, stigmatization and shame may be behind potentially bigger numbers of infected or ill people who do not come forward for treatment or care. Attitudes of their kin care providers need to be addressed as well as those of the official health care providers.

## Introduction

When HIV/AIDS was identified, different psychological responses accompanied it and the community looks to it as a social phenomenon (1). The control of spread of HIV takes concerted efforts at both national and international levels (2). Education is an important component of preventing the spread of HIV. The education about HIV/AIDs that guided by colleagues and families were succeeded in improving knowledge and behaviors to prevent the spread of this disease (3).

Fighting HIV/AIDS needs concerted efforts toward changing cultural and traditional beliefs, myths, misconceptions and modes of learned behavior (4).

Some agencies such as the Joint United Nations Program on HIV and AIDS (UNAIDS) in 1994 was established to prevent the disease and care for patients with this disease (5).

Addressing the topic of HIV/AIDS as a potentially alarming endemic is relatively new in the Middle East at large, Jordan being a case in point. Due to seemingly cultural and religious considerations, reluctance has hindered deliberate discussion to deal with the pandemic through an adequate and effective educational and advocacy program.

Jordan has a very low incidence HIV epidemic, both amongst the overall population and amongst significant populations at higher risk of HIV exposure. AIDS was detected in Jordan in 1986, the overall number of HIV positive cases identified within the period (1986–2011) is 847 (29% Jordanians, 71% foreigners). The overall number of HIV positive cases recorded in 2010 and 2011 is 36 (78% males, 22% females).

Sexual Contact is remaining the main mode of HIV transmission, accounting for almost 65% of HIV-positive persons. Until the end of Dec 2011, 99 Jordanian people living with HIV had died of AIDS (6). In some societies, women were supposed as the main transmitters of HIV/AIDS (1).

Nearly, 26% of infections were due to blood and blood products; these appear to have occurred early in the epidemic. Injected drug use and mother-to-child transmission together accounted for 6% of HIV/AIDS cases. Nearly, 6 % of all infections were attributable to unknown modes of transmission (6). As a result, the National Jordanian AIDS Program was established through the General Directorate for Primary Health Care. A National Committee was then formed, with representation from all health sectors, to monitor all national activities related to AIDS, through a number of epidemical, laboratory, educational and treatment committees. The National Committee ensured the provision of health care services to patients (carriers and those infected) and has intensified screening of blood transfusion; blood transactions in Jordan are centralized subject to 100% and mandatory testing. A number of campaigns were also launched to increase citizens’ awareness of how the disease is contracted and on the means of protection. However, sexuality remains forbidden especially for youth. Hence, education for all sectors of society, especially to youth, becomes extremely important. Due to the limited ways by which HIV can be transmitted, increasing the awareness of the public regarding HIV and their knowledge of HIV and AIDS will greatly assist in overcoming increasing infection rates.

Little is known as to whether parents discussed sexuality and STDs with their children. Furthermore, little is known as to whether adults themselves had reasonable knowledge of safe sexual behavior and STDs. Thus, this study aimed to assess:

- Current attitudes of parents, teachers, and students towards informing children about HIV/AIDS,
- Attitudes concerning the ‘proper’ age that parents believe their children should learn about AIDS,
- Possible differences in attitudes relating to the gender of the child and what they feel male and female children should learn,
- Ideas about the most ‘adequate’ person/institution to be responsible for provision of HIV/AIDS education.

## Materials and Methods

### Setting and Sample

This study was conducted in Amman, Jordan in April 2015 on a sample of school students, university students, schoolteachers and parents utilizing a stratified random sampling as follows:

School students sample: (N=60)

1. Two governmental schools (one for girls and one for boys) and one private school (for both girls and boys) were randomly selected from each educational directory (north, east, south and west of Amman).
2. From each school, five students were randomly selected (one student from each grade starting from the 8^th^ grade).

School teachers sample: (N=60)

1. Five teachers were randomly selected from the same schools.

Parents sample: (N=60)

1. Thirty mothers and thirty fathers residing in a sample of houses selected, using the house-to-house method, form the schools’ neighborhood were approached.

*Mothers and fathers approached had at least one teenager (or young adult), son or daughter.

University students’ sample: (N=60):

Thirty male students and thirty female students enrolled at the different faculties at the oldest university in the country were selected. The first male student and the first female student, who enjoyed a Jordanian nationality, met at each faculty were asked to participate in the study.

### Survey Instrument

The investigators based on an extensive review of related literature and included two parts developed a questionnaire; the first part pertained to demographic data and the other part included questions on substantive components addressing the opinions regarding HIV/AIDS education. The questionnaire was designed in two forms: one targeting youth (school and university students), and the other targeting community members (parents and teachers). A group of experts in the subject tested the instrument for validity. The instrument was pilot-tested on a random sample that was not included in the actual survey. Modifications were done according to the results of the pilot study. Internal consistency reliability using Cronbach’s alpha reliability was 0.61. Split half technique was also used to test reliability and its value was 0.68.

### Data Collection

Permissions to conduct the research were asked following the appropriate channels. Meetings with key people were conducted to secure cooperation. Four research assistants were recruited and trained on the proper methods of data collection; the coding system was used to secure anonymity of the study sample, basic ethical issues, and data cleaning and entry for one week. The informed consent of each selected subject was obtained, for the children; the permission from their parents was obtained. The research assistants collected the data from each respondent on an individual basis. The time needed to fill each questionnaire was between 15 and 20 min.

### Data analysis

The SPSS program (Chicago, IL, USA) was used for data analysis, simple and inferential statistics were done.

## Results

All groups asserted the importance of HIV/AIDS education and awareness raising for all. This percentage was higher than 97% for all groups (except teachers). Teachers’ percentage was significantly lower but still high at 90%.

There were significant differences among the various groups regarding the main source of HIV/AIDS education (Table 1). The highest significant difference was in relation to school and family (*P*=0.001 for both). School students and university students were the least to agree that school is the main source of information (35%, 58% respectively), while the highest was for male teachers at 83% followed by mothers (80%), fathers (73%) and female teachers (70%).

**Table 1:** The main source of information relating to HIV/AIDS

**Source**	**University Students**		**School Students**		**Mothers**		**Fathers**		**Male Teachers**		**Female Teachers**		**Total**		**Sig.**
	**No.**	**%**	**No.**	**%**	**No.**	**%**	**No.**	**%**	**No.**	**%**	**No.**	**%**	**No.**	**%**	
School	35	58	21	35	24	80	22	73	25	83	21	70	148	62	0.00*
Family	15	25	16	27	15	50	19	63	20	67	14	47	99	41	0.00*
Friends	9	15	16	27	5	17	5	17	8	27	3	10	46	19	0.33
Physician	19	32	21	35	12	40	14	47	10	33	11	37	87	36	0.81
Television	26	43	35	58	16	53	22	73	22	73	17	57	138	58	0.04*
Radio	5	8	15	25	7	23	9	30	8	27	4	13	48	20	0.08
Papers/Magazines	16	27	31	52	14	47	12	40	11	37	7	23	91	38	0.04*
Others	22	37	16	27	5	17	4	13	5	17	4	13	56	23	0.05*
Don't know	3	5	0	0	0	0	0	0	1	3	1	3	5	2	0.36

Significant at (*P*=0.05)

Only 20% of all respondents reported that the radio is the main source of information on HIV/AIDS with no significant differences, 58% reported the television with a significant difference of *P*=0.04.

Overall, 82% of respondents believed that HIV/AIDS education should be integrated into the different disciplines/subjects of the school curriculum. On the other hand, 44% believed that it has to be a specific subject in the school curriculum. No significant differences among the various groups of respondents were reported to either alternative.

Regarding the respondents’ opinions related to the age at which AIDS education should be started in schools; only 6% of total respondents believed that HIV/AIDS education should be integrated into school curriculum before age 12, while 49% of respondents believed the right age to be between 12 and 15 yr and 43% between 16 and 19 yr of age. The differences were statistically significant for answers second and third age groups (*P*=0.03 and 0.001, respectively) where 60% of university students compared with 38% of school students believed it should be integrated between ages 12 and 15. The percentages of the adult respondents were also higher for the parents (67% mothers and 50% fathers) compared with the teachers (40% male and 37% female teachers). In the third suggested age group, 53% of both female and male teachers believed that HIV education should be integrated into school curriculum after age 15; 57% of school students also believed. Of 84% of total respondents believed that HIV/AIDS education should be part of university curricula. There were no significant differences among the various groups; however, it was highest for fathers at 96% followed by female teachers at 93%.

Reasons for opposing the integration of HIV/ADIS education in school curricula are reflected in (Table 2). The table shows that 32% of the respondents opposed because they believed it is related to the topic of sex and as such may raise sex curiosity among students. They thought that HIV/AIDS education would prompt children and adolescents to purse sexual relations at an early age. Another 16% opposed because they believed that the topic is related to drugs and may activate students to try them. There were no significant differences between the various groups in this regard.

**Table 2:** Reasons for opposing the integration of HIV/ADIS education in school curricula

**Reasons**	**University students**		**School students**		**Mothers**		**Fathers**		**Male teachers**		**Female teachers**		**Total**		**Sig.**
	**No.**	**%**	**No.**	**%**	**No.**	**%**	**No.**	**%**	**No.**	**%**	**No.**	**%**	**No.**	**%**	
Raises sex curiosity	22	37	22	37	13	43	6	20	6	20	8	27	77	32	0.20
Family issue	2	3	3	5	0	0	0	0	0	0	0	0	5	2	0.36
Against religious beliefs	2	3	4	7	4	13	2	7	1	3	1	3	14	6	0.48
Raises curiosity for drugs	13	22	11	18	5	17	0	0	4	13	6	20	39	16	0.17

Significant at *P*=0.05

Almost nobody believed that HIV/AIDS education should be restricted to boys only. Of 65% respondents believed that HIV/AIDS should not be discussed with girls and boys before puberty. There were no significant differences between the groups in this regard; however, it was highest for the university students (77%). On the other hand, 85% of the sample total respondents believed that HIV/AIDS education at an early age arms people with knowledge about the dangers of HIV/AIDS and prepares them to avoid being prey to infection. About 82% of total respondents believed that there should be television programs on HIV/AIDS, targeting each age group specifically.

Of 75% of all respondents believed that schoolteachers would make the best educators on HIV/AIDS. Although the percentage of schoolchildren concerning teachers was substantial (63%) yet it was consistent with their response in relation to the main source of information regarding HIV/AIDS whereby only 35% of school students said that the school was the main source of their information.

## Discussion

The difference between students and adults may be interpreted as youth, especially the school age students are too shy to admit sex education in school, or that they did not believe that the school could provide the information they needed. Nevertheless, it is interesting to see that adults, in varying degrees, believed in the role of school as means of HIV education. This finding is in contrast with the International Planned Parenthood Federation study (7) conducted in Greece where many parents did not consider teachers as being the most appropriate people to inform their children or to undertake sexual education in schools.

Regarding the family, adults seem to put more emphasis on the family being the main source of information on HIV/AIDS. This finding is similar to another study, whereby as many as 76.4% of parents reported that they were the most appropriate people to inform their children on health issues and sexuality (7). However, while this opinion of Greek parents is related to the fact that 91.4% of them believed that they are able to discuss issues relating to sexuality with their children, in Jordan it may be assumed that adults still consider HIV and sexuality issues as taboo and hence would rather restrict them to private discussions within the family. The parents’ attitude about family being the source of sex education to their children is in contrast with the children’s attitudes, in both Jordan and Greece. Additionally, most parents do not discuss sexual matters, traditional rules, and taboos freely with their children as they still attach much value to their cultural traditions that are not so open on sex matters (8). Furthermore, in South Africa 36% reported never or hardly ever communicating about sexuality with parents, other adult family members, and teachers, respectively (9). Adolescent females preferred to receive sexuality information from their mothers, while males preferred to receive sexuality information from their fathers.

Thirty-six percent of respondents believed that physician should be the main source of HIV/AIDS education. This result is not unexpected, as physicians had been always considered an authority in health matters.

While only 20% of respondents reported that radio is the main source of information, 58% reported the television with a significant difference of *P*=0.04. This may be a reflection of city people depending less and less on radio in light of their mobile lifestyle that television is taking over as a more interesting medium. Likewise, university students (37%) and school students (27%) reported that the internet is considered a source of information on HIV/AIDS. This may be related to the fact that the internet is becoming an essential part of university and school students as compared to adults some of whom are computer illiterate.

Eighty-two percent of all respondents believed that HIV/AIDS education should be integrated into the different disciplines/subjects of the school curriculum. On the other hand, 44% believed that it has to be a specific subject in the school curriculum. The Jordanian public opinion had been set in a certain direction, considering that HIV/AIDS education is the subject of the discussion, without voicing the matter of sex education. According to the IPPF study, the issue of whether sexual education in high schools in Greece should be a separate course or part of a broader curriculum on health education and health behavior remains unresolved despite long-running discussions (7).

Only 6% of respondents believed that HIV/AIDS education should be integrated into school curriculum before age 12, while around half of respondents believed the right age to be between 12 and 15 yr and 43% between 16 and 19 yr of age. While the majority of university students compared with 38% of school students believed it should be integrated between ages 12 and 15. The percentages of the adult respondents were also higher for the parents (67% mothers and 50% fathers) compared with the teachers (40% male and 37% female teachers). In the third suggested age group, 53% of both female and male teachers believed that HIV education should be integrated into school curriculum after age 15; 57% of school students also believed so. This result is incongruent with the results of the IPPF study that revealed students as saying that they should have been introduced to sex education at the elementary level.

The majority of respondents believed that HIV/AIDS education should be part of university curricula. This result may be attributed to the fact that at this stage, students are considered to be mature enough to comprehend the concepts relating to sexuality and sexually transmitted diseases and to be more in need for such information.

Almost nobody believed that HIV/AIDS education should be restricted to boys only. In the Greek study, parents discussed different sets of topics with girls and boys. The study in Jordan, examined general sexuality topics, only HIV/AIDS. HIV education made a greater difference in women’s self-reflection of their behavior than it did with men (10).

Totally, 82% of respondents believed that there should be television programs on HIV/AIDS, targeting each age group specifically. The percentages were highest among male teachers (90%) and lowest among the female teachers (73%). In the case of Greece, mass media was believed to be the main source of information in health issues with TV being the primary source. However, many parents (80%) believed that today in Greece the mass media do not adequately cover health issues and suggested that there should be more TV programs about health and related matters. On the other hand, in the case of Uganda, cartoons have significant as an educational resource for HIV/AIDS education (11).

Although the percentage of schoolchildren concerning teachers was substantial (63%) yet it was consistent with their response in relation to the main source of information regarding HIV/AIDS whereby only 35% of school students said that the school was the main source of their information. The results were incongruent with those of the IPPF study where only 7.9% of the parents mentioned teachers as the right people to give such information. They believed that the most appropriate person to teach these subjects were a health specialist (40.7% of parents) or a doctor (37.1% of parents).

The study was limited to the capital and major city in Jordan. As such, it is not conclusive and certainly calls for a more in-depth complementary study. The scope of inquiry can be expanded to include further topics such as attitudes of health care personnel, and teachers assigned the task of educating students. Furthermore, religious beliefs did not seem to hamper the introduction of HIV/AIDS education in schools or to the public.

## Conclusion

The study in Jordan misses people’s attitudes towards the actually infected or HIV carriers. This has serious implications as to how society will need to deal with patients, provide them with the services they need, without being stigmatized and rejected by society, their family members, peers, or the health care providers.

As HIV/AIDS is a scary matter to all, stigmatization and shame may be behind potentially bigger numbers of infected or ill people who do not come forward for treatment or care. Attitudes of their kin care providers need to be addressed as well as those of the official health care providers.

## Ethical considerations

Ethical issues (Including plagiarism, informed consent, misconduct, data fabrication and/or falsification, double publication and/or submission, redundancy, etc.) have been completely observed by the authors.
